# Notes and News

**Published:** 1900-08-25

**Authors:** 


### Notes and News.

The rumour was spread abroad that a case of the plague had appeared in Berlin, but this is now denied.

It is proposed to close the wards of University College Hospital whilst the new buildings are being taken into occupation.

Dr. Alan Herbert, who for 28 years has served as physician to the Hertford British Hospital in Paris, has been presented with an address by the former and present medical officers of the hospital.

In Vienna a baby-rearing exhibition has been recently held, in which all modern methods for bringing up young children in a healthy manner was shown. Such an exhibition in connection with one of our annual exhibitions at Earl's Court, or elsewhere, would prove most instructive to English mothers, and be very popular besides.

The majority of the staff of the Portland Hospital have just returned from South Africa after having rendered splendid service. The Portland Hospital was the first civilian hospital to go out. Major Kilkelly, R.A.M.C., who was military commandant, Mr. A. Bowlby, and Mr. E. Calverly have transferred their services to other hospitals.

The presentation of a bust of Queen Victoria as a Jubilee memorial has been made to the City of London Hospital for Diseases of the Chest by Sir Mancherjee Bhownaggree. The Lord Mayor unveiled the bust, which is the work of Mr. A. E. L. Rost. The hospital is amongst those which have adopted the open-air treatment for consumption, and new balconies were opened on the same occasion by the Lord Mayor.

It is well known that rats are largely responsible for the dissemination of the plague, but it is suspected that it is by means of the vermin which infect them that they become so dangerous. Dr. Munro gives a word of warning to those instituting a crusade against them, advising them to pour paraffin over rats when killed and then to remove them by means of tongs. He also points out that ships should be fumigated before coming into port, that the rats may not come ashore.

The last returns from Sydney show that the plague is practically at an end there.

We learn that the Princess Christian—as well as the Queen, the Prince and Princess of Wales, and the Duke and Duchess of York—has withdrawn her patronage of the Royal Military Benevolent Fund at 93, Queen's Gate.

We are informed that the Grand Prix in Class 16, Medicine and Surgery, at the Paris Exhibition, has been awarded to Messrs. Down Bros. for their exhibit of surgical instruments and aseptic hospital furniture.

An unfortunate accident occurred at Charing Cross Hospital, where a fire was occasioned by the upsetting of some beeswax and turpentine set to melt on a stove. The clothing of the servant who removed the melting material became ignited, and she was so badly burned that death ensued. The fire was promptly put out.

It is held by many that the Boers have sometimes had a legitimate excuse for firing upon field hospitals and ambulances flying the ordinary Red Cross flag, which is not always distinguishable at firing distance. To obviate mistakes, Mr. Charles Maggs has invented an apparatus to supersede the flag. It consists of four metal wings fixed on a central axis; on both sides of each wing half a red cross is painted, so that in all aspects a large red cross is visible. The apparatus folds flat, and is easily carried.

We greatly regret to have to record the death of Sir William Stokes, M.D., F.R.C.S.I., which took place at Pietermaritzburg last Saturday. In December last he was appointed a consulting surgeon to the forces, and ever since his arrival in South Africa he has been engaged in hospital work among the wounded. Sir William Stokes was born in Dublin in 1839, and was the son of the late Dr. William Stokes, Regius Professor of Medicine in the University of Dublin, and Surgeon-in-Ordinary to the Queen. Sir William Stokes received his medical education at Dublin, Berlin, Vienna, Paris, and London. He was President of the Royal College of Surgeons of Ireland, Professor of Surgery to the same, President of the Pathological Society of Ireland (of which he was in earlier days a Gold Medallist), and, like his father, he was Surgeon-in-Ordinary to the Queen.

The annual report of the Public Control Department of the London County Council covers a large amount of ground, and is full of most interesting facts. The fact that no case of rabies has been reported in London since June, 1898, is alone a matter for congratulation, and shows that progress is certainly being made. The increasing use made of petroleum is another noticeable fact. It is pointed out that so far efforts made to secure the flash point at 100 deg. Fahrenheit have been unavailing, and the number of lamp accidents continue to be very large. Seeing that the committee have failed to enforce the regulation they desire, they might well make an effort in another direction which would help the end they have in view. They might endeavour to secure the prohibition of the sale of any receptacle for the consumption of petroleum made of glass or china. The risk of the falling lamp is small when made of metal. The report cites a good many instances of efforts made on behalf of the public good, which have been unavailing so far.

